# The incentivized drug information services among community pharmacists: a multi-centre cross-sectional study in Indonesia

**DOI:** 10.1017/S1463423624000537

**Published:** 2025-01-24

**Authors:** Muh. Akbar Bahar, Mersa N. Kausar, Khairunnisa Khairunnisa, Ivan S. Pradipta

**Affiliations:** 1 Department of Pharmacy, Faculty of Pharmacy, Hasanuddin University, Makassar, Indonesia; 2 Occupational Health Regional Public Hospital West Java Provincial Government, Bandung, Indonesia; 3 Drug Utilization and Pharmacoepidemiology Research Group, Center of Excellence in Higher Education for Pharmaceutical Care Innovation, Universitas Padjadjaran, Bandung, Indonesia; 4 Faculty of Pharmacy, Universitas Sumatera Utara, Medan, Indonesia; 5 Department of Pharmacology and Clinical Pharmacy, Faculty of Pharmacy, Universitas Padjadjaran, Bandung, Indonesia

**Keywords:** community pharmacists, drug information services, remuneration

## Abstract

**Background:**

Community pharmacists should provide qualified drug information services for the rational use of medicine in community. However, there is no standard professional incentive for the service in Indonesia. This study aimed to assess drug information services with incentives and its associated factors among community pharmacists in Indonesia.

**Method:**

A multi-centre cross-sectional study was conducted among community pharmacists in Medan City, Bandung City, Bandung Regency, and Makassar City. A validated online self-administered questionnaire was used to collect data on pharmacists’ demographics, pharmacy characteristics, and drug information provision practices. Multivariate logistic regression was applied to identify factors associated with incentivized drug information services.

**Results:**

A total of 639 community pharmacists participated, with representation from Medan (21.9%), Bandung City (20.8%), Bandung Regency (26%), and Makassar (31.3%). Most respondents were female (79%) with a median age of 31 years (IQR: 9). Only 12% of pharmacists reported receiving incentives for providing drug information services. Factors significantly associated with receiving incentives included being male (OR: 2.04, 95% CI: 1.16–3.58), aged 20–30 years (OR: 3.25, 95% CI: 1.10–9.58), working over 40 hours per week (OR: 2.30, 95% CI: 1.16–4.58), working in a chain pharmacy (OR: 2.08, 95% CI: 1.18–3.67), and having an onsite physician practice (OR: 1.72, 95% CI: 1.04–2.85).

**Conclusion:**

Limited number of community pharmacists received an incentive for drug information services. The development of a remuneration system for drug information services can be considered to enhance the quality of pharmaceutical care services in the community setting.

## Introduction

The community pharmacist is one of the fundamental healthcare providers in the community (Kennie-Kaulbach *et al.*, 2012). They are considered easy to access and among the most dependable healthcare (Crossley 2019; Tsuyuki *et al.,* 2018). In the pharmaceutical care era, the professional roles of pharmacists have substantially developed to not only focus on product-related services but also provide patient-centred care (PCC) via direct patient–pharmacist interaction (Olson *et al.,* 2021). In PCC, patients should be actively involved in deciding the best therapy that meets their personalized needs. Therefore, providing drug information becomes one of the main elements in PCC, in which pharmacists, as drug experts, are expected to provide this service (Ghaibi *et al.,* 2015). Delivering drug information to patients is essential in ensuring the safety, effectiveness, and appropriate medicine use to achieve the expected clinical outcomes (Alfian *et al.,* 2021; Mino-León *et al.*, 2015).

However, in some developing countries, pharmacists still spend most of their time in their traditional role of drug compounding and dispensing instead of doing cognitive services such as providing drug information (Bhagavathula *et al.,* 2014; Fathelrahman *et al.,* 2016). Several barriers have been reported to hinder the implementation of the pharmaceutical care model in these developing countries, from human resources-related problems to unsupported healthcare systems, including remuneration system (Puspitasari *et al.,* 2015; Scahill & others 2014).

The provision of drug information is an essential and standard pharmacy service that is professionally and legally expected to be provided by community pharmacists in Indonesia (Andayani & Satibi 2016; Faller 2022). However, Indonesia is still among those countries where its community pharmacists struggle to adapt to their new role as clinical healthcare providers (Herman & Handayani 2015; Hermansyah *et al.,* 2020).

Lack of remuneration is one of the commonly cited problems to hamper the implementation of pharmaceutical care services in Indonesia (Hermansyah *et al.,* 2018a). Consequently, the pharmaceutical care service cannot be optimally provided, leading to an unrecognized role among health care providers and the community.

A previous study indicated that the current remuneration system in Indonesia has not adequately supported the provision of these cognitive clinical activities (Hermansyah *et al.,* 2021). The same study reported that although more than 80% of the community pharmacists perceived that they should have received a compensation for their services, only less than 50% received incentives for delivering patient care services such as medication review and drug information services (Hermansyah *et al.,* 2021).

The limited study has analyzed remuneration issues in drug information services provided by community pharmacists. Therefore, we aimed to identify the proportion and characteristics of Indonesian community pharmacists compensated for providing drug information services and to investigate which characteristics independently influence this practice. Identifying these determinants is expected to help the decision-makers develop a proper intervention to accelerate the optimal pharmaceutical care service in community settings.

## Methods

### Study design and setting

A multi-centre cross-sectional study was conducted from July to October 2021 to assess the characteristics of Indonesian community pharmacists (*Apoteker Komunitas in Indonesia*) compensated economically for providing drug information services. A self-developed, valid, and anonymous questionnaire was distributed among community pharmacists in four different regions representing the western (Medan city), central (Bandung city and regency), and eastern parts (Makassar city) of Indonesia. All participants filled out an informed consent before participating in this study. Ethical approval was obtained from ’the ethics committee of Universitas Sumatera Utara No. 599/KEP/USU/2021. The research was conducted in accordance with the Declaration of Helsinki.

### Instrument and variables

The data collection instrument was part of a valid and reliable questionnaire developed to study the ‘knowledge, attitude, and practice of Indonesian community pharmacists in tuberculosis patient detection’ (Pradipta *et al.,* 2022). The questionnaire items were carefully developed based on a thorough literature study and were reviewed by professionals, including a community pharmacist and a pharmacy technician. To improve its content, the questionnaire was pre-tested with a sample of 32 individuals who were typical of the target community. Please see our prior article for a thorough overview of the whole validation procedure (Kausar *et al.* 2023). The questionnaire comprises two types of questions: multiple choice and short answer questions. The latter includes inquiries regarding age, years of working experience, weekly working hours, and staff count. These variables underwent a grouping process based on theoretical considerations and their distribution patterns, aiming to enhance comparability among different segments within the dataset. We defined sociodemographic and pharmacy characteristics as the exposure variables, while remunerated drug information provision practice was defined as the outcome variable in this study.

### Participants

This study used purposive sampling, which included only community pharmacists working in the pharmacy (*Apotek in Indonesia*) with at least 6 months of experience. The 6 months were used to ascertain that the community pharmacist regularly provided drug information services during their daily practice. Pharmacists working in the community health centre, called *Puskesmas in Indonesia,* were excluded. Puskesmas is the primary health care centre and is managed by the local government. The pharmacists working in Puskesmas are civil servants and their payments are regulated by the government. Therefore, they are not eligible for this study. Considering the COVID-19 pandemic, the researchers distributed the questionnaire online (via social media such as WhatsApp, Facebook, and Telegram) and offline (direct visits to the community pharmacies) with the help of the local pharmaceutical professional organizations in each district.

### Sample size

According to the formula proposed by Green (1991) (*n* > 50 + 8m, where ‘m’ is the number of potential determinants), the sample size required for this study (nine variables) should be more than 122 patients (Green, 1991).

### Data analysis

Descriptive statistics were used to summarize pharmacists’ and pharmacies’ essential characteristics. Categorical data were displayed in the form of numbers and percentages. Meanwhile, normally distributed continuous data were presented as a mean and standard deviation. Skewed distributed variables were presented as median and interquartile range (IQR). Bivariate logistic regression was applied to assess the relationship between the independent (characteristics of pharmacists and their pharmacies) and dependent (remunerated drug information services) variables. A *p*-value <0.25 was used as a threshold to include potential variables in the multivariate analysis. Adjusted odds ratio (OR) and 95% confidence interval (CI) were obtained for each determinant using multivariate logistic regression analysis. A *p*-value of 0.05 indicated a statistically significant association between the determinants and outcome.

## Results

We collected information from 639 community pharmacists, comprising pharmacists from Medan City (21.9%), Bandung City (20.8%), Bandung Regency (26%), and Makassar City (31.3%). Most of the participants were female (78.6%) with a median age of 31 years old (IQR: 9). Some parts of the respondents held master’s or Ph.D. degrees (13.8%), and most of them (70.6%) had five years or less working experience. We identified that 35.8% of the participants had a 20-hour or less duration of work in a week, and most of the pharmacists (87.2%) only handled one pharmacy. Most of the pharmacies (77.8%) were independent pharmacies, and 43% of the pharmacies were embedded with medical doctor’s practice. About 39% of the pharmacies had three to four staff, and around 37% had five or more staff. Characteristics of the subjects are presented in Table 1.

**Table 1.** Characteristics of pharmacists and pharmacies (*n* = 639)

Variables	Values
**Sex**	
Women (*n*, %)	502 (78.6%)
Men (*n*, %)	137 (21.4)
Age in years (median, IQR)	31 (9)
**Age in years**	
20–30 (*n*, %)	297 (46.5)
31–40 (*n*, %)	249 (39.0)
>40 (*n*, %)	93 (14.6)
**Level of academic education**	
Bachelor (*n*, %)	551 (86.2)
Master or PhD (*n*, %)	88 (13.8)
**Years of practice**	
≤5 years (*n*, %)	451 (70.6)
6–10 years (*n*, %)	110 (17.2)
>10 years (*n*, %)	78 (12.2)
**Working week in hours**	
≤20 (*n*, %)	229 (35.8)
21–40 (*n*, %)	215 (33.6)
>40 (*n*, %)	195 (30.5)
**Numbers of workplaces**	
1 Pharmacy (*n*, %)	557 (87.2)
≥2 Pharmacies (*n*, %)	82 (12.8)
**Type of pharmacy**	
Independent pharmacy (*n*, %)	497 (77.8)
Chain pharmacy (*n*, %)	142 (22.2)
**The presence of physician practice**	
No (*n*, %)	364 (57.0)
Yes (*n*, %)	275 (43.0)
**Number of staff in the pharmacy**	
1–2 persons (*n*, %)	151 (23.6)
3–4 persons (*n*, %)	252 (39.4)
≥5 persons (*n*, %)	236 (36.9)
**Location of pharmacy**	
Medan City (*n*, %)	140 (21.9)
Bandung City (*n*, %)	133 (20.8)
Bandung Regency (*n*, %)	166 (26.0)
Makassar City (*n*, %)	200 (31.3)
**Remunerated drug information services**	
No (*n*, %)	564 (88.3)
Yes (*n*, %)	75 (11.7)

We identified that only 75 participants (12%) received compensations for drug information services to the patients. Most of them were female (69.3%) and were young age (60% were in the age category of 20–30). Our data showed that 78.7% had equal to or less than five years of working experience, and most subjects worked more than 40 hours per week. In terms of pharmacy characteristics, we identified that 82.7% were responsible for only one pharmacy, and about 59% worked in an independent pharmacy. In total, 56% of these community pharmacists worked with a physician in their pharmacies; lastly, more than 80% worked with more than two personnel (Table 2).

**Table 2.** Potential determinants of incentivized drug information services

Variables	Remunerated Drug Information Services		OR (95% CI)	*P* value
	Yes (*n* = 75)	No (*n* = 564)		
**Sex**				
Women (*n*, %)	52 (69.3)	450 (79.8)	Ref.	
Men (*n*, %)	23 (30.7)	114 (20.2)	1.75 (1.03–2.97)	0.04
**Age in years**				
20–30 (*n*, %)	45 (60)	252 (44.7)	3.97 (1.39–11.36)	
31–40 (*n*, %)	26 (34.7)	223 (39.5)	2.59 (0.88–7.65)	
>40 (*n*, %)	4 (5.3)	89 (15.8)	Ref.	0.02
**Level of academic education**				
Bachelor (*n*, %)	62 (82.7)	489 (86.7)	Ref.	
Master or PhD (*n*, %)	13 (17.3)	75 (13.3)	1.37 (0.72–2.61)	0.34
**Years of practice**				
≤5 years (*n*, %)	59 (78.7)	392 (69.5)	1.81 (0.75–4.34)	
6–10 years (*n*, %)	10 (13.3)	100 (17.7)	1.20 (0.42–3.45)	
>10 years (*n*, %)	6 (8.0)	72 (12.8)	Ref.	0.26
**Working week in hours**				
≤20 (*n*, %)	15 (20.0)	214 (37.9)	Ref.	<0.01
21–40 (*n*, %)	23 (30.7)	192 (34.0)	1.71 (0.87–3.37)	
>40 (*n*, %)	37 (49.3)	158 (28.0)	3.34 (1.77–6.30)	
**Numbers of workplaces**				
1 Pharmacy (*n*, %)	62 (82.7)	495 (87.8)	Ref.	
≥2 Pharmacies (*n*, %)	13 (17.3)	69 (12.2)	1.50 (0.79–2.88)	0.22
**Type of pharmacy**				
Independent pharmacy (*n*, %)	44 (58.7)	453 (80.3)	Ref.	
Chain pharmacy (*n*, %)	31 (41.3)	111 (19.7)	2.88 (1.74–4.76)	<0.01
**The presence of physician practice**				
No (*n*, %)	33 (44.0)	331 (58.7)	Ref.	
Yes (*n*, %)	42 (56.0)	233 (41.3)	1.81 (1.11–2.94)	0.02
**Number of staff in the pharmacy**				
1–2 persons (*n*, %)	14 (18.7)	137 (24.3)	Ref.	0.52
3–4 persons (*n*, %)	30 (40.0)	222 (39.4)	1.32 (0.68–2.58)	
≥5 persons (*n*, %)	31 (41.3)	205 (36.3)	1.48 (0.76–2.84)	

The bivariate analyses indicated that sex, age, working week, number of workplaces, type of pharmacy, and the presence of physician practice were potential determinants of compensated drug information services (*p* < 0.25) (Table 2). Furthermore, multivariate logistic regression revealed that male (OR: 2.04 [95% CI: 1.16–3.58]), age group of 20–30 years old (OR: 3.25 [95% CI: 1.10–9.58]), more than a 40-hour working week (OR: 2.30 [95% CI: 1.16–4.58]), chain pharmacy (OR: 2.08 [95% CI: 1.18–3.67]), and the presence of physician practice (OR: 1.72 [95% CI: 1.04–2.85]) were significantly associated with the incentivized drug information practice (Table 3).

**Table 3.** Independent determinants of incentivized drug information services

Variables	OR (95% CI)	*P* value
**Sex**		
Women (*n*, %)	Ref.	
Men (*n*, %)	2.04 (1.16–3.58)	0.01
**Age in years**		
20–30 (*n*, %)	3.25 (1.10–9.58)	0.03
31–40 (*n*, %)	2.61 (0.87–7.84)	0.09
>40 (*n*, %)	Ref.	
**Working week in hours**		
≤20 (*n*, %)	Ref.	
21–40 (*n*, %)	1.52 (0.76–3.04)	0.24
>40 (*n*, %)	2.30 (1.16–4.58)	0.02
**Numbers of workplaces**		
1 Pharmacy (*n*, %)	Ref.	
≥2 Pharmacies (*n*, %)	1.11 (0.56–2.19)	0.77
**Type of pharmacy**		
Independent pharmacy (*n*, %)	Ref.	
Chain pharmacy (*n*, %)	2.08 (1.18–3.67)	0.01
**The presence of physician practice**		
No (*n*, %)	Ref.	
Yes (*n*, %)	1.72 (1.04–2.85)	0.04

## Discussion

In Indonesia, the provision of drug information has been reported to increase patients’ compliance with their medication, decrease drug-related problems, and improve clinical outcomes (Athiyah *et al.,* 2021; Darmawan *et al.,* 2020; Sauriasari & Sakti 2018). According to ‘Government Regulation Number 51 of 2009 on Pharmacy Practice’, one of the mandatory services that a community pharmacist must provide to customers is drug information (Andayani & Satibi 2016). However, an economic barrier has hampered the implementation of this service daily. The current remuneration systems in Indonesia for community pharmacist is predominantly in the form of fixed monthly payments and mainly without any extra incentives for cognitive clinical care (Hermansyah *et al.,* 2018a, 2021). In this present study, we found that only 12% of the participants were compensated with an additional payment for providing drug information services. These results indicated that despite the vital role of the pharmacist in providing drug information services, compensation for their work has been minimal to support such a role.

Previous research reported that community pharmacists’ drug information services in Indonesia is generally low (Herman & Susyanty 2012; Listyorini *et al.,* 2021). Furthermore, the pharmacist generally provides this drug information services as a free service (Hermansyah *et al.,* 2021). If they receive economic compensation, the commission often comes from the patients, with the pharmacist billed for the service to be included in the dispensing cost. However, this system might not be feasible in the long term to support the viability of pharmacist services since it is difficult to demonstrate to patients the benefits and values underlying the payment (Hermansyah *et al.,* 2021).

We found that male pharmacists were more often to receive financial benefits when providing drug information services than female pharmacists. This disparity might be related to differences in communication skills between the sexes, though the data are conflicting. A study assessing the communication skills of pharmacy students indicated that female students were more hesitant and less confident than their male counterparts (Ubaka & Ukwe 2019). Similar findings were observed among medical students, where female students appeared less confident and more doubtful, often underestimating their competence as health practitioners compared to male students (Blanch *et al.,* 2008). Other studies found contradictory results. Female pharmacy students have shown a better attitude towards learning communication skills than male students (Svensberg *et al.,* 2018). Additionally, a literature review indicated that female doctors were more involved in patient-centred communication than male doctors (Roter and Hall 2004). However, a study conducted in Australia found no correlation between gender and a patient-centred communication style, although this study had a small sample size of 20 pharmacists (Chong *et al.,* 2014). The mixed results might be influenced by factors such as culture, study type, and sample size, underscoring the need for further research on this issue. Moreover, it is possible that female pharmacists provide drug information services but they do not charge their services. Our questionnaire simplified the inquiry to identify who had remunerated drug information services and who did not, thus we lack data on the proportion of pharmacists who share drug information, categorized by gender, without charging for the service.

The next characteristic associated with compensating drug information services is age. The younger the community pharmacists, the higher chance they get compensation for providing drug information services. A previous study reported that younger pharmacists provide more detailed information about drugs to consumers than older pharmacists (Svarstad *et al.,* 2004). It might be related to access to drug information resources. Younger pharmacists were reported to mostly use electronic drug information knowledgebase (Carvajal *et al.,* 2013). Consequently, they can provide faster and more complete information than older pharmacists using the printed version of drug information resources (Carvajal *et al.,* 2013). Patients were reported to be willing to pay for the counselling session if they found their pharmacists were helpful and provided complete information (AlShayban *et al.,* 2020).

In this study, we found that more than 70% of the participants had less than five years of working experience. Surprisingly, we found that this particular variable did not exhibit a significant independent association with the provision of incentivized drug information services. This observation prompts further examination into potential underlying factors contributing to this lack of correlation. One possible explanation is that recent pharmacy graduates might have received updated training programs emphasizing the importance of providing drug information services. In this scenario, formal education and training could compensate for the lack of practical experience.

We also found that the longer the duration of working at the pharmacy in a week, the higher the chance of pharmacists receiving additional incentives for their drug information services. Pharmacists with a long duration of work in a week can have flexible time to provide individualized drug counselling to patients. Therefore, patients might feel the importance of the service. It was reported that if patients could get counselling services without difficulties and with proper time duration, they would be willing to pay for the service (AlShayban *et al.,* 2020). Another finding from this study was that pharmacists working at the chain pharmacy were more likely to get financial benefits in providing drug information services than those working in the independent pharmacy. Herman & Handayani (2015) reported that pharmacists working in an independent pharmacy are less prepared to provide pharmaceutical care services than those working in a chain pharmacy (Herman & Handayani 2015). Indonesian chain pharmacists mostly operate under centralized management systems with relatively big capital (Athiyah *et al.,* 2019). Therefore, they can implement standard operating procedures for delivering high-quality care to their customers (Athiyah *et al.,* 2019). They also have a good training system to provide pharmacists with clinical skills in pharmaceutical care services, including drug information. Consequently, they provide a reasonable compensation for each cognitive service provided by their pharmacists (Hermansyah *et al.,* 2018b).

Lastly, we also found that pharmacists who work with physicians more often receive remuneration for their drug information services than those who work independently. It might be related to peer pressure created by the collaborative work between the two health professionals. Ebeling *et al.* (2012) reported that effort provision increases when peer pressure is present (Ebeling *et al.* 2012). Therefore, the pharmacist might provide good cognitive service, including drug information provision, when working with physicians. Ultimately, patients may voluntarily choose to pay for such high-quality services.

Some limitations are worth mentioning. First, this study might not represent the overall condition of Indonesian community pharmacists since this study used a purposive sampling method. To minimize the limitation, we tried to sample the community pharmacists from three big cities in the western, central, and eastern parts of Indonesia to get a comprehensive picture of the practice. We also sampled pharmacists from a suburban area to increase the coverage of our study. Second, we only found a small proportion of pharmacists having extra payment for delivering drug information. However, it needs to be interpreted cautiously. In Indonesia, there is a low rate of pharmacists’ attendance in the community pharmacy, with less than 25% of them working full-time hours, leaving pharmacy-related services being delivered by the pharmacy technicians (Dominica *et al.,* 2016; Hermansyah *et al.,* 2018b; Kartinah *et al.,* 2015). Therefore, it might be that some participants in this study did not get extra incentives simply because they did not deliver drug information services since they were mostly absent in the pharmacy. This study found that about 36% of the participants worked 20 hours or less in a week. Third, we found that male pharmacists were more likely than their female counterparts to receive financial incentives for providing drug information services. However, it is important to acknowledge the sex disparity among our participants, with female pharmacists comprising 78.6% of the sample. Nevertheless, it is pertinent to highlight that the pharmacy profession in Indonesia is predominantly female. According to the 2019 national analysis of Indonesia’s pharmacy workforce, it was documented that 78% of pharmacists in Indonesia were women (Meilianti *et al.,* 2022). This sex distribution also aligns with findings from previous studies conducted in Indonesia, which have consistently reported a comparable proportion of female pharmacists (Kusuma *et al.,* 2023; Rendrayani *et al.,* 2023; Wathoni *et al.,* 2023). Fourth, we distributed the questionnaire online due to the COVID-19 pandemic. Consequently, we may not have reached a representative number of older pharmacists with limited smartphone usage capabilities. We only encountered approximately 15% of pharmacists aged over 40 years old. Therefore, our sample might be potentially biased towards younger pharmacists. Another possible reason is that most of older Indonesian pharmacists (>36 years old) work in industry and government institutions, while younger pharmacists are predominantly employed in patient-facing roles such as hospitals, community pharmacies, and clinics (Meilianti *et al.,* 2022). Fifth, there was a possibility that the COVID-19 pandemic might influence the ability of community pharmacists with only 6 months of experience in delivering drug information services. However, in our sample, only 9% of participants had approximately 6 months of experience working as community pharmacists. Among these, only eight pharmacists reported providing remunerated drug information services. Due to the small sample size for this subset, we cannot draw definitive conclusions from our current data. Future studies with larger sample sizes and a focus on the pandemic’s impact on pharmacists’ roles and capacities, especially for those early in their careers, would be beneficial to provide more comprehensive insights. Sixth, the causality between the exposure and outcome should be interpreted cautiously since this cross-sectional study did not consider the time difference between exposure and outcome.

This study has several strengths. This is the first study exploring the rate of incentivized drug information services among community pharmacists in Indonesia. Additionally, this study is also the first to identify factors that independently influence this practice. Indonesian consumers in the community pharmacy expect their pharmacists to provide more time and detailed information during drug consultation to support self-medication (Alfian *et al.,* 2016). However, the general provision of cognitive services by community pharmacists has been hampered by the absence of payment for the service (Hermansyah *et al.,* 2021). The economic compensation of pharmaceutical care activities has been identified as one factor facilitating changes in pharmacy practice (Roberts *et al.,* 2008). Therefore, the results of this study can be beneficial for stakeholders such as the government, insurance companies, pharmacist’s associations, and pharmacy owners as foundational data to deliver personalized interventions to enhance the delivery of optimal pharmaceutical care services in community settings.

Several future studies could further enhance our understanding of drug information services among community pharmacists in Indonesia. First, qualitative studies could explore the perceived barriers and facilitators for pharmacists who are not compensated for providing these services. Second, a cross-sectional study could be designed to investigate the knowledge, skills, perceptions, and attitudes of community pharmacists regarding drug information services. Lastly, cost-effectiveness studies could be developed to integrate these services into the health insurance system, ensuring a sustainable pharmacist remuneration system and high-quality drug information services. Coverage of health insurance in the drug information services may be beneficial to improve drug information services in Indonesia.

## Conclusion

In conclusion, this study found that only a few Indonesian community pharmacists receive compensation when providing drug information services. Several characteristics independently associated with this compensated drug information services are sex (male), age (younger pharmacists), type of pharmacy (chain pharmacy), and the presence of a medical doctor in the pharmacy.
